# Chilaiditi Syndrome Complicated by Cecal Perforation in the Setting of Scleroderma

**DOI:** 10.1177/2324709618803387

**Published:** 2018-09-29

**Authors:** Tagore Sunkara, Prashanth Rawla, Krishna Sowjanya Yarlagadda, Gerald A. Baltazar, Vinaya Gaduputi

**Affiliations:** 1 The Brooklyn Hospital Center, Clinical Affiliate of the Mount Sinai Hospital, New York, NY, USA; 2 Memorial Hospital of Martinsville and Henry County, Martinsville, VA, USA; 3 SBH Health System, New York, NY, USA

**Keywords:** Chilaiditi syndrome, colon perforation, abdominal pain, scleroderma

## Abstract

Chilaiditi syndrome is a very rare disorder characterized by abdominal pain due to the entrapment of the colon between the liver and the diaphragm. However, it is rare to have bowel perforation as a complication of this syndrome with only 2 cases reported to date. In this article, we present the case of a 56-year-old woman with medical history of scleroderma who presents with abdominal pain and was found to have colonic perforation from Chilaiditi syndrome. She was also incidentally found to have cecal adenocarcinoma. Sometimes abdominal pain in patients with Chilaiditi syndrome may be more than benign and calls for increased attention from clinicians regarding this.

## Introduction

Chilaiditi sign is the finding of bowel in between the liver and right diaphragm^1^ and is usually an incidental finding on chest or abdominal radiographs.^2^ When it is associated with symptoms of entrapment, it is called Chilaiditi syndrome.^3^ Symptoms include abdominal pain, distension, bloating, nausea, vomiting, flatulence, change in bowel habits, and rarely substernal pain, shortness of breath, or arrhythmias.^2^ Very rarely is it associated with bowel perforation, and there are only 2 cases reported to date.^4,5^ Here we present the case of a 56-year-old woman who presented with Chilaiditi syndrome and cecal perforation and was coincidentally found to have an ascending colon adenocarcinoma.

## Case Report

A 56-year-old woman with a past medical history of scleroderma, chronic constipation, and hypertension presented to the emergency room with generalized abdominal pain associated with multiple episodes of vomiting. Pain was described as 5/10 in intensity, localized in the right lower quadrant with no exacerbating or relieving factors. Vomiting was nonbloody and nonbilious. She has also been constipated more than usual for the past week. On presentation, vital signs were normal except for mild tachycardia of 104. Physical examination is significant for right lower quadrant tenderness and decreased bowel sounds. Initial blood count and basic metabolic panel were normal but the lactic acid on presentation was 4.4 mmol/L.

**Figure 1.**
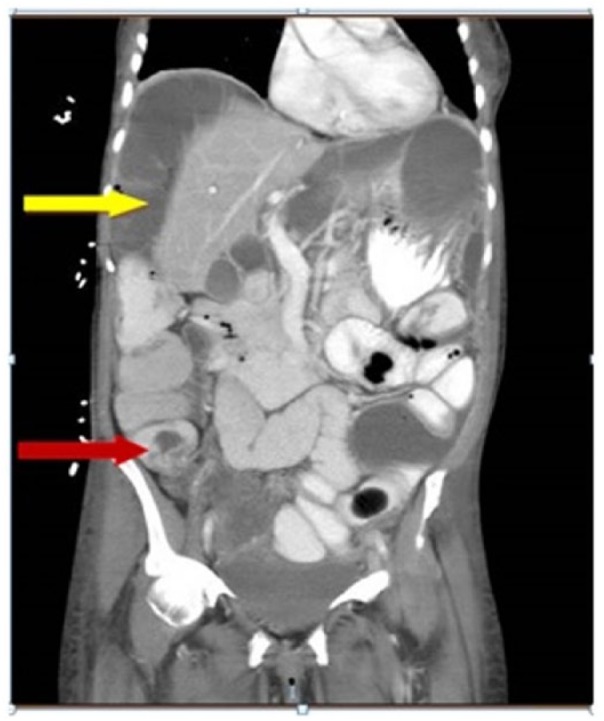
Computed tomography scan of abdomen showing Chilaiditi sign with interpositioning of colonic loop in between the liver and diaphragm (yellow arrow), cecal wall thickening (red arrow), and free fluid.

**Figure 2.**
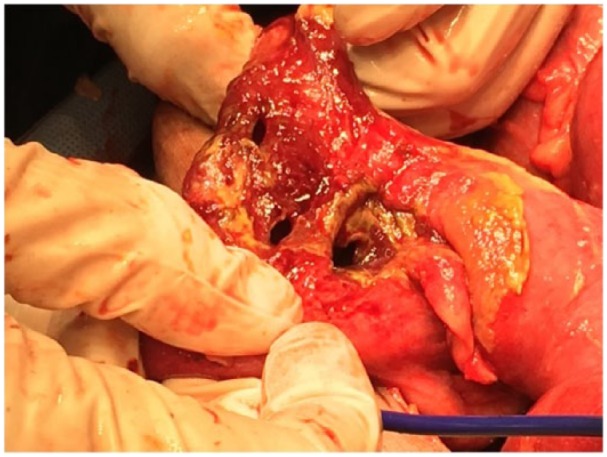
Exploratory laparotomy showing ischemia and perforation in the cecum.

A computed tomography (CT) scan revealed multiple loops of large bowel positioned between the liver and the right diaphragm indicative of Chilaiditi syndrome, cecal wall thickening (Figure 1), multiloculated pelvic abscess with droplets of air suggestive of peritonitis, and segmental distension of several loops of distal small bowel concerning for ileus or partial obstruction. The patient underwent CT-guided drainage of the pelvic abscess with return of 600 cc of purulent material after which the patient was started on intravenous vancomycin, piperacillin/tazobactam, and metronidazole. Over the next 2 days, drain output was increased gradually along with spike in white blood cell count. Repeat CT scan showed worsening of the pelvic fluid collection as well as development of new distant fluid collections in the anterior and outer left abdomen. The patient underwent exploratory laparotomy with abdominal washout and right hemicolectomy. Operative findings included feculent peritonitis and necrotic cecum with perforations (Figure 2). Pathology of the specimen reported moderately differentiated adenocarcinoma with invasion into pericolonic adipose tissue. Perforation in the cecum was likely related to a combination of factors. There was a segment of normal intervening colonic mucosa between the nonobstructing cecal mass and the cecal perforation. The lumen was widely patent at the time of perforation and therefore we believe that compromised wall integrity from underlying scleroderma and luminal compression from coexisting Chilaiditi syndrome played a role in the perforation in the cecum. There were no tumor elements at the site of perforation on histology. The patient was discharged with an end ileostomy on day 7 and was later seen in surgery clinic in 2 weeks with improvement in symptoms. The patient is scheduled to follow-up with hematology oncology.

## Discussion

Chilaiditi sign was described in 1865 but was named in 1910.^6^ As mentioned above, the presence of symptoms in patients with the sign is called the Chilaiditi syndrome. It is incidentally found in 0.025% to 0.28% of chest or abdominal plain radiographs and 1.18% to 2.4% of abdominal CT scans.^2^ It is predominantly seen in males and elderly population.^6^

Risk factors for the syndrome include intestinal causes (abnormal motility, long colon/mesentery, no peritoneal attachments, and congenital malposition), hepatic cause (reduced liver size, laxity of hepatic suspensory ligaments), diaphragmatic causes (abnormally high diaphragm), and other causes (ascites, increased abdominal fat, pregnancy, and aerophagia).^2^

There is also an association of Chilaiditi syndrome in literature to scleroderma described in these 2 case reports.^7,8^ However, none of them presented with alarming symptoms of perforation. Colonic volvulus was frequently reported presentation in various case reports and it needed emergency surgery.^6,7,9,10^

To date, there are only 2 cases of perforation reported with Chilaiditi syndrome.^4,5^ One is a 54-year-old male with no past medical history, while another one is an 81-year-old male with past medical history of coronary artery disease, diabetes mellitus, hypertension, dyslipidemia, diverticulosis, and rheumatic disease. Both patients were treated with surgical management and had good outcomes.

Our patient with a history of scleroderma-associated constipation presented with abdominal pain and was diagnosed with cecal perforation. He was found to have incidental Chilaiditi syndrome on the CT scan. After exploratory laparotomy he was also found to have an ascending colonic adenocarcinoma. Two main factors to be considered here that could have contributed to perforation are weakened bowel wall from scleroderma and acute and chronic constipation from scleroderma. We confirmed that the mass did not cause perforation.

In conclusion, not all episodes of abdominal pain in patients with Chilaiditi syndrome are benign, and clinicians should maintain a high index of suspicion in ruling acute bowel catastrophes like ischemia or perforation.
